# The modern structurator: increased performance for calculating the structure function

**DOI:** 10.1140/epje/s10189-021-00146-2

**Published:** 2021-12-02

**Authors:** Mojtaba Norouzisadeh, Mohammed Chraga, Giovanni Cerchiari, Fabrizio Croccolo

**Affiliations:** 1 E2S UPPA, CNRS, TOTAL, LFCR UMR5150, Universite de Pau et des Pays de l’Adour, Anglet, France; 2 Institut für Experimentalphysik, Universität Innsbruck, Technikerstrasse 25, 6020 Innsbruck, Austria; 3 Present Address: Institut des Sciences de la Terre d’Orléans, CNRS, Université Orléans, 1A rue de la Férollerie, 45071 Orléans, France

## Abstract

The autocorrelation function is a statistical tool that is often combined with dynamic light scattering (DLS) techniques to investigate the dynamical behavior of the scattered light fluctuations in order to measure, for example, the diffusive behavior of transparent particles dispersed in a fluid. An alternative approach to the autocorrelation function for the analysis of DLS data has been proposed decades ago and consists of calculating the autocorrelation function starting from difference of the signal at different times by using the so-called structure function. The structure function approach has been proven to be more robust than the autocorrelation function method in terms of noise and drift rejection. Therefore, the structure function analysis has gained visibility, in particular in combination with imaging techniques such as dynamic shadowgraphy and differential dynamic microscopy. Here, we show how the calculation of the structure function over thousands of images, typical of such techniques, can be accelerated, with the aim of achieving real-time analysis. The acceleration is realized by taking advantage of the Wiener–Khinchin theorem, *i.e.*, by calculating the difference of images through Fourier transform in time. The new algorithm was tested both on CPU and GPU hardware, showing that the acceleration is particularly large in the case of CPU.

## Introduction

Dynamic light scattering (DLS) techniques have been used for decades to obtain information about the dynamical behavior of a variety of samples spanning from soft matter physics to biology [1]. The main idea of DLS is to measure the intensity of the light scattered by a transparent sample at a given angle and statistically analyze its fluctuations in time in order to obtain information on the motion of the components inside the sample. For example, DLS analysis of the Brownian motion of particles dispersed in fluid allows measuring their diffusion coefficient and then, ultimately, their size distribution thanks to the Stokes–Einstein relation between the particles’ mobility and their size. The quantity classically obtained in DLS instruments is the autocorrelation function, i.e., the direct output of “correlators” that compute the scalar product of the intensity signal coming from the light detector by the same quantity at different delay times. An alternative approach to the autocorrelation function has been proposed several decades ago and consists in computing the structure function. The structure function is obtained by analyzing the autocorrelation of the differences of the signal at different times [2]. At the same time, it was proposed to develop “structurators” in place of the more widely known correlators [3]. With the spread of pixelated detectors, imaging techniques like dynamic shadowgraphy, dynamic Schlieren [4–9], and differential dynamic microscopy (DDM) [10–12] have taken advantage of the use of the structure function because of its improved robustness for data analysis in terms of rejection of background signal deriving from steady-state and slow-drift noise sources as compared to the autocorrelation function approach [2, 13]. This is due to the intrinsic nature of the structure function that is based on the difference of signal elements of increasing time delay so that any spurious signal changing on times longer than the utilized time delay is subtracted. By using the spatial Fourier analysis, these imaging techniques allow scientists to investigate the temporal evolution of a sample at the different length scales present in a set of images recorded at different times [13]. For this reason, they have gained popularity, especially in the field of soft matter physics. In fact, the combination of the robustness of the structure function analysis applied to simple and/or already available optical setups has allowed them to be used both in traditional laboratories [10–12], and in orbiting experiments on the ISS [14–16] for investigating the dynamics of rather different samples ranging from colloidal particles [10] to bacteria [17], but also from biological cells [18] to density fluctuations in and outside thermal equilibrium [4, 19], and many others, as also witnessed by several review articles [12, 13, 20, 21].

As stated, the structure function approach can be combined with imaging techniques, thus requiring an optical system like transmitted light microscopy [10], fluorescence-based microscopy [22], dark-field imaging [23] to acquire series of images. The series of images should be processed by custom-made software to compute the structure function, as defined by Schultz-Dubois and Rehberg [2] and later implemented to Schlieren [8] and Shadowgraphy [8] and optical microscopy [10, 24]. However, a rapid evaluation of the structure function is fundamental to achieve real-time analysis in laboratory conditions and may play a crucial role in the utilization of such an approach in industrial and commercial applications. The available software programs calculate the structure function in different ways. Some process the images by calculating the differences between pairs of images first, and then evaluate the bi-dimensional fast Fourier transforms (FFT) of the differences [11]. In other cases, they first compute the FFT of the images and then calculate the differences in Fourier space [25].

Since the number of images that can be acquired and the number of pixels therein have considerably increased in the latest two decades, the computational load to evaluate the structure function has increased consequently. In the meantime, also the computational capabilities of modern computers have grown, but a major breakthrough in reducing the computation time of the structure function was achieved when researchers started to implement the calculation on graphics processing units (GPU) [22, 25]. The implementation of this computational task on GPU allowed a decrease in the computational time by a factor of 10–30, thereby reducing the data analysis time from several hours to a few tens of minutes.

In the present article, we present a different route to calculate the structure function of the image series taking advantage of the Wiener–Khinchin theorem  [26, 27]. The calculation is performed by using the Fourier transform in time rather than by calculating differences of spatial FFTs. This approach enables a further optimization step and allows us to compute the structure function faster than state-of-the-art existing software. We obtain a considerable speed up of the calculation time, particularly when GPU acceleration is not available.

The article is organized as follows. First, we provide an example of application by means of Shadowgraph images that are later utilized to test the software performances. Then, we discuss our method for calculating the structure function and compare it with state-of-the-art algorithms [25]. Finally, we discuss the results and provide conclusions.

## Test case: shadowgraph observation of density fluctuations

In this section, we describe a free diffusion experiment obtained by carefully layering two miscible fluids where the denser one is placed at the bottom of the container, so to obtain a gravitationally stable condition. The fluid system is investigated by shadowgraphy, i.e., an optical technique able to measure density fluctuations within the fluid in terms of series of images $I_m$ from which one can extract the density fluctuation structure function by means of the DDA algorithm.

In the classical implementation of the DDA algorithm  [2, 4, 8, 10], the structure function is calculated by first evaluating the differences among all pairs of images and then by computing the power spectra of those differences, and finally, by averaging the power spectra over all the pairs of images acquired with the same time delay. This procedure can be defined as follows:

1 $$\begin{aligned} d\left( m\right) =\frac{1}{N-m}\sum _{n=m}^{N-1}|F_{xy} \left( I_{n-m}-I_{n}\right) |^2 \; , \end{aligned}$$

where the indices *n* and *m* run from 0 to $N-1$ and $F_{xy}$ indicates the bidimensional FFT of the images in space. The absolute value operation “$|\ldots |$” is intended for every wave vector component of the FFT.

The initial condition is prepared in two steps: First, we introduce pure water by completely filling a glass cylindrical cell (Hellma, 120-OS-20); second, we slowly inject the glycerol and water solution (20 % w/w) until reaching half of the cell. By using this procedure, we obtain a two-layer sample in which the two miscible liquids are separated by a vanishing horizontal interface and are stabilized by the gravitational force while the only mechanism relaxing the concentration gradient with time is mass diffusion. The dissolving concentration gradient provides a non-equilibrium condition that amplifies the spontaneous velocity fluctuations within the fluid [28]. This results in the appearance of non-equilibrium fluctuations at all wavelengths that can be visualized by means of the Shadowgraph setup as done in several publications [4].

For shadowgraph observation, the cell is illuminated by a collimated plane-parallel beam obtained by using a super-luminous diode (Superlum, SLD-MS-261-MP2-SM) with a wavelength of $\lambda =(675 \pm 13)$ nm and propagating along the vertical axis. The light propagates through the sample and the density fluctuations induce local fluctuations of the refractive index that scatter the light field. A charged coupled device (CCD) records the interference between the primary laser beam and the light scattered by refractive index fluctuations inside the fluid. We acquired sets of $N=$ 2000 images $I_n$ of $512\times 512$ pixels at the frame rate of 25 Hz.

**Fig. 1:**
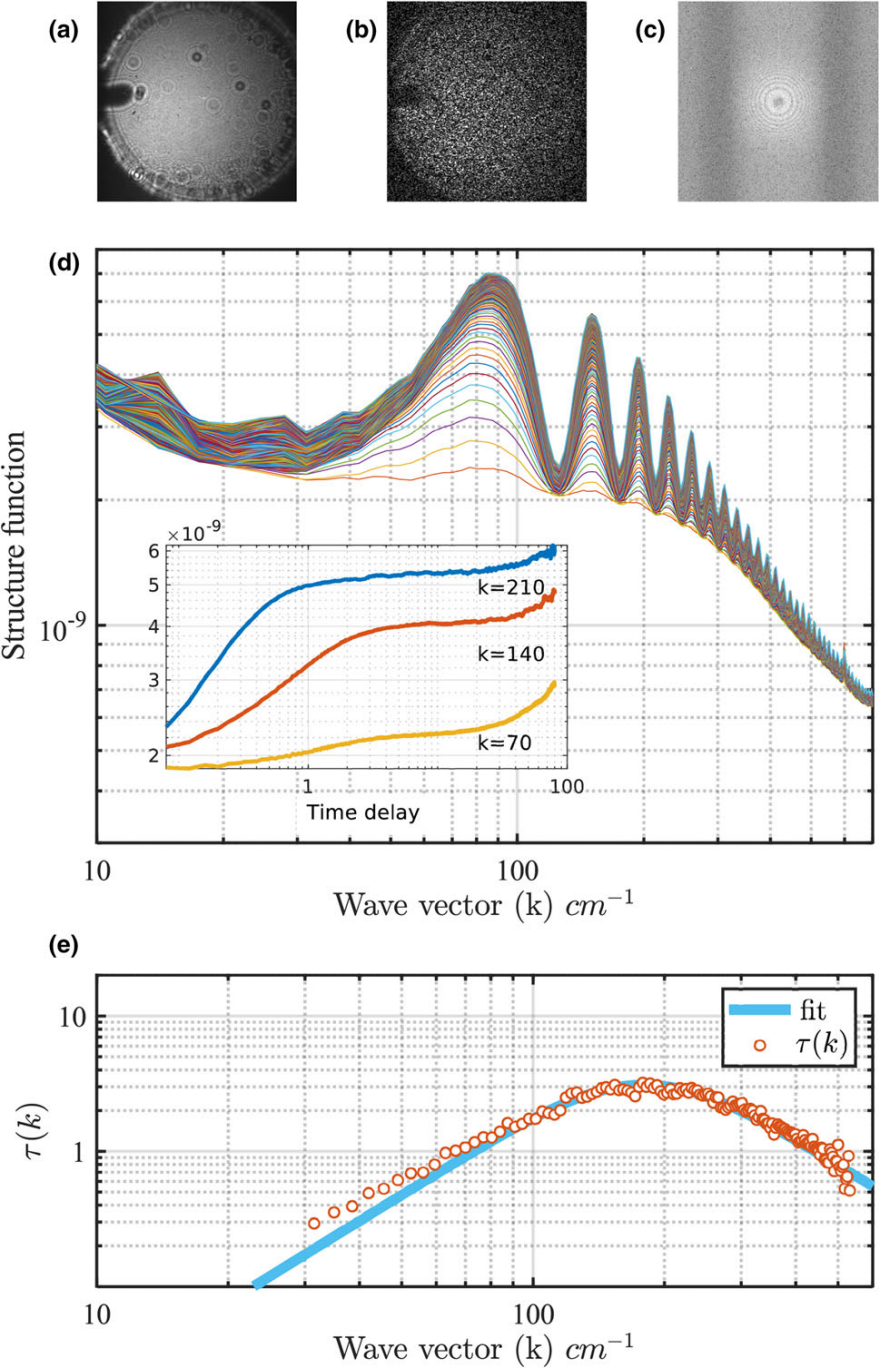
Sample data adopted for the tests reported in this article. The data corresponds to a measurement of the non-equilibrium concentration fluctuations in a free-diffusion experiment. **a** Sample image consisting of $512\times 512$ pixels, the size of the image in the real space is 19 mm. **b** Difference of two images taken 4 s apart. **c** Structure function averaged over 1000 difference of image pairs with 4 s time delay. **d** Angular average of bi–dimensional structure functions like the one shown in **c** for different time delays. **d**–inset Structure function as a function of the time delay for three different wave vectors. **e** Time decay $\tau (k)$ as obtained by fitting the structure functions with a model function containing a single exponential decay

In Fig. 1 we show: (a) a typical Shadowgraph image $I_{n}$, (b) a typical image difference $\left( I_{n-m}-I_{n}\right)$ with enhanced contrast to make the tiny density fluctuations visible, and (c) its bidimensional power spectrum $|F_{xy} \left( I_{n-m}-I_{n}\right) |^2$ displayed in logarithmic scale. The two images considered for the difference were taken 4 s apart so that the signal is uncorrelated for most of the wave vectors, and the structure function has already reached its maximum. In panel (d), the azimuthal average $\langle d(m)\rangle _\phi$ of the structure functions are shown for many delay times *m*. The inset of panel (d) shows the structure function as a function of the delay time for three selected wave vectors. The strong oscillations as a function of the wave vector are related to the shadowgraph transfer function as described in literature [9]. The structure function increases as a function of the delay time at any wave vector and can be analyzed to investigate the diffusive behavior of concentration fluctuations during the diffusion process. The structure function is modeled as detailed in the literature [5], by providing a suitable model for the intermediate scattering function, which in the present case is a single exponential decay. The fitting procedure thus provides a measurement of the time decay $\tau (q)$ for any wave vector *q* as shown in panel (e). The right part of the plot shows the typical $1/(Dq^{-2})$ behavior of concentration non-equilibrium fluctuations from which one can extract the value of the mass diffusion coefficient, like it has been performed for thermodiffusion experiments [5]. The left part of the plot shows the effect of gravity on the decay times of concentration non-equilibrium fluctuations already reported in several ground-based experiments [8]. The latest part of such analysis is out of the scope of the present paper and will be published in a separate work.

## Different approaches to the structure function

The calculation of the structure function involves evaluating differences, FFTs and averages that can be performed efficiently on a GPU as parallel operations [22]. This approach can be optimized by exploiting the linearity of the FFT and the available hardware memory as described in ref. [25]. The calculation of Eq. 1 can be approached via a two-step algorithm. First, all FFTs $\tilde{I}_n = F_{xy}I_n$ of the images $I_n$ are calculated and stored in the local memory. Second, each matrix *d*(*m*) is evaluated by averaging differences of the FFTs of images $(\tilde{I}_{n-m}-\tilde{I}_n)$ rather than FFTs of image differences $F_{xy}(I_{n-m}-I_n)$ exploiting the linearity of the FFT operation. This approach reduces the number of operations to be performed because the matrices $\tilde{I}_n$ can be used several times for different *d*(*m*). Thus, for *N* images, the number of FFTs to be computed is reduced from $O(N \times N)$ to *O*(*N*). While this optimization allows reducing the number of FFTs, the overall algorithm has a global computational complexity of $O(N \times N)$. We see this from Eq. 1 because there are as many time delays *m* as images, and for each *m* the matrix *d*(*m*) is obtained via a sum over $(N-m)$ images.

In this work, we present a new approach to reduce the global computational complexity of the algorithm to $O(N \times \log _2(N))$ by using the Wiener–Khinchin theorem [26, 27], which states that, for a stationary random process, the autocorrelation function can be calculated by the power spectrum (in time) of the process.

We expand the square modulus operation of Eq. 1 in the following way:

2 $$\begin{aligned} d\left( m\right)&=\frac{1}{N-m}\sum _{n=m}^{N-1}\left( |\tilde{I}_{n-m}|^2+|\tilde{I}_{n}|^2-2\text {Re}\left( \tilde{I^*}_{n-m}\tilde{I}_{n}\right) \right) \; , \end{aligned}$$

where the symbol “$*$” indicates complex conjugation. In the sum, the first term $|\tilde{I}_{n-m}|^2$ is the average of the first $(N-m)$ spatial power spectra, while the second term $|\tilde{I}_n|^2$ is the average of the last $(N-m)$ spatial power spectra. Both terms have a computational complexity of $O\left( N\right)$. The last term, identified by the product $\tilde{I}^*_{n-m}\tilde{I}_n$, is the autocorrelation function of the image FFTs. The autocorrelation is the only term in Eq. 2 which has computational complexity of $O\left( N\times N\right)$. By applying the Wiener–Khinchin theorem [26, 27], the autocorrelation function can be evaluated via the power spectrum in the temporal frequency Fourier space. The advantage of computing the autocorrelation function via the Fourier transform in time is given by the speedup provided by the FFT algorithm allowing to reduce the computational complexity from $O\left( N \times N\right)$ to ($O\left( N\times \log _2\left( N\right) \right)$) [29].

## Performance analysis

To compare the new algorithm with other available reference software [25, 30–32], we developed a new software program that implements the algorithm described in ref. [25] and the new algorithm on CPU and GPU hardware, for a total of four execution modes. Further comparisons with other software are presented in “Appendix D” showing that the method reported in [25] was already one of the fastest approaches to calculate the structure function before the present work. To distinguish the two algorithms, we will refer to the method reported in ref. [25] as WITHOUT_FT and the technique discussed in this article as WITH_FT, where the label FT stands for Fourier transform in time. Both methods calculate the final result in two steps. The first step is common and consists of calculating and storing the FFTs of the images in the available free memory: RAM for the CPU versions and G-RAM (global RAM) for the GPU implementations. In the second step, the wave vectors are analyzed independently according to the different schemes. If the wave vector data exceeds the capacity of the available memory, both algorithms split the job into several groups at the price of recalculating the image FFTs several times (see “Appendix C” for more details). The program is written in C++11 and CUDA v.10.2 with graphical support of the OpenCV 3.0 library. We tested the program with the Fourier transform libraries CUFFT (version provided in CUDA v.10.2) for GPU execution and FFTW 3.3.3 [33] for the CPU implementations. The code was compiled with MS compiler v120 and the compiler of CUDA v.10.2 in Visual Studio 2019. The program was executed on a machine with the following specifications:

- CPU: Intel®$$\hbox {Core}^{\mathrm{TM}}$$ i9-9880H,
- 32 GB DDR4 RAM,
- Graphic card: NVIDIA Quadro RTX 4000 with 8GB of dedicated G-RAM memory,
- 512 GB SSD drive—PCIe, performance class 40.

**Fig. 2:**
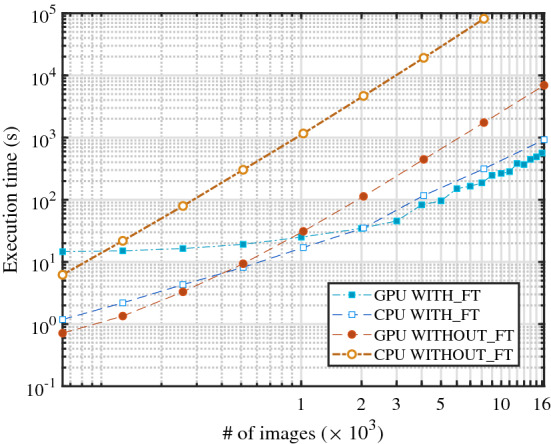
Execution time as a function of the total number of images for images of $512\times 512$ pixels. The data points corresponding to the WITH_FT algorithm have square markers. The data points corresponding to the WITHOUT_FT algorithm have circular markers. The markers have colored filling for the GPU modes, and have white filling for the CPU modes

Test images of $512\times 512$ pixels were taken from the experiment described in Sect. 2. For other sizes, synthetic images were generated with $n\times n$ pixels having 16 bit depth, similar to the real images. In our tests, we considered image sets composed by maximum $2^{14}=16 384$ images, and we limited the execution time of the program to less than $10^5$ s.

In the first test, we ran all the algorithms on CPU and GPU with images composed of $512\times 512$ pixels. For comparison, we made use of 8 GB of RAM for executing the program on the CPU. In this way, the CPU and the GPU could access the same amount of RAM and G-RAM, respectively. The execution times of the program are presented in Fig. 2, in which the times for all four execution modes are plotted as a function of the number of images used for the test. As expected from the results reported in ref. [25], the WITHOUT_FT algorithm executes more than 30 times faster on GPU than CPU. The GPU hardware is also faster than the CPU in executing the WITH_FT algorithm, but the speed-up factor never exceeds a factor of two. We see that the WITH_FT scheme is faster than the WITHOUT_FT method when the image number processed in one run of the program is larger than $\sim 1000$. If the condition $N\gtrsim 1000$ is met, both CPU and GPU versions of the WITH_FT algorithm execute quicker than the GPU–WITHOUT_FT implementation, reaching a maximum speed-up factor of 10–12 for 16384 images.

**Fig. 3:**
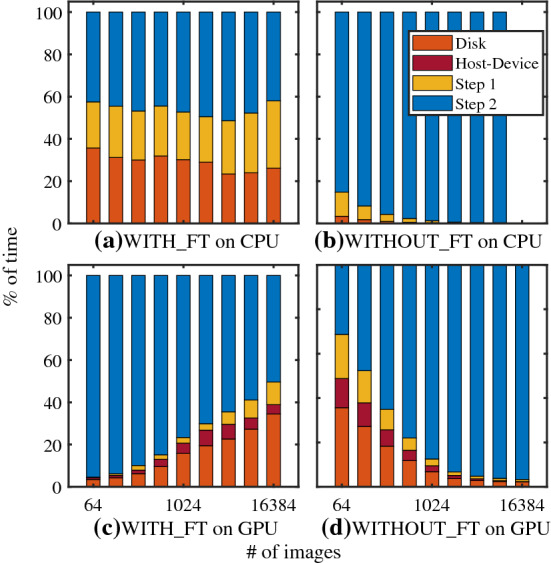
Fractional execution time of the different tasks of the program as a function of the total number of images. The length of the colored bars represents the fractional time spent by the program to execute the different operations: *disk* IO, *host–device* data transfers, *step 1* and *step 2*. In this test, we used images of $512\times 512$ pixels. The first row (graphs (**a**), **b**) presents the fractional times spent in CPU mode and the second row (graphs **c**, **d**) in GPU mode. The first column shows the fractional times of the WITH_FT algorithm (graphs **a**, **c**) and the second column of the WITHOUT_FT algorithm (graphs **b**, **d**). Data of the CPU-WITHOUT_FT version for 16384 images are no reported because the total execution time was exceeding $10^5$ s

Figure 3 presents the fractional time spent by the program in the four modes to compute the images’ FFTs (*step one*), process the time sequences (*step two*) and perform memory IO operations (*disk* and *host-device*). The IO operations named *host-device* include the data transfers between the RAM and the G-RAM, and it exists only in the GPU implementations. In the figure, we normalized the fractional times by the total execution time to highlight the different workloads for executing each part of the program. As a function of an increasing number of images, the workload of step two compared to the other operations remains balanced in the CPU-WITH_FT implementation, and it reduces in the GPU-WITH_FT implementation. Conversely, the WITHOUT_FT algorithm spends more fractional time during the second step as the number of images increases both in the CPU and the GPU modes. Combining the information of Figs. 2 and 3, we see the advantage of the new implementation applied to the problem of calculating the structure function. The WITH_FT algorithm is faster than the WITHOUT_FT scheme for a large number of images as a consequence of the reduction in computational complexity in processing the time sequences of the wave vectors.

**Fig. 4:**
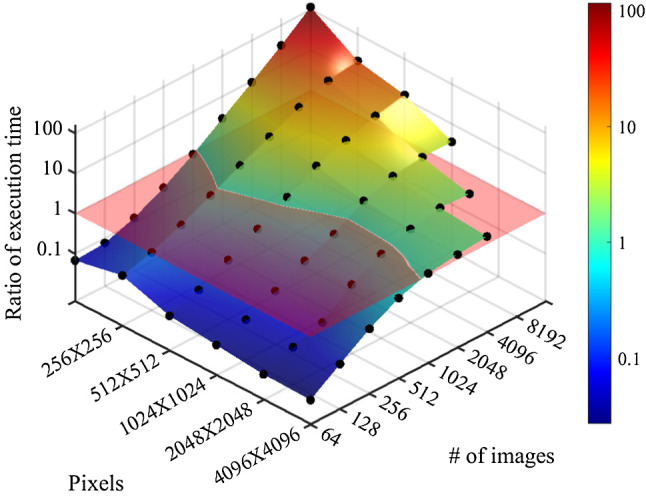
Ratio of execution times on GPU of the WITHOUT_FT against the WITH_FT algorithm as a function of different numbers and sizes of images. The transparent red plane marks the condition in which both algorithms process the images within the same time

In a second test, we analyzed the execution performance of the GPU-WITH_FT and GPU-WITH OUT_FT algorithms for squared images of different sizes. Figure 4 presents the ratio of execution times between the GPU-WITH_FT execution over GPU-WITHOUT_FT execution for a different number of images and different image sizes. In analogy to the $512\times 512$ pixels example, the WITH_FT method is faster than the WITHOUT_FT technique for more than $\sim 500-1000$ images. The red plane in the figure marks the condition in which both algorithms complete execution in the same amount of time. We notice that small image sizes obtain a larger speedup gain as compared to large images. For example, images composed of $128\times 128$ pixels obtain up to a $\sim 100$ speed-up gain in the execution time, against only $\sim 4$ obtained with images composed of $1024\times 1024$ pixels. In fact, the number of pixels per image affects the load of data transfer operations and FFT of the images in two ways. First, calculating the bidimensional FFT requires more time for images composed of many pixels. Second, the FFTs are calculated several times if the wave vector components of all the images exceed the available memory. Therefore, at processing images composed of many pixels, both the WITH_FT and WITHOUT_FT algorithm must spend a large fraction of time preparing the time sequences before their analysis. Considering for example the WITH_FT at processing 16384 images, the first step and memory IO operations occupy 44% of the execution time with images composed of $1024\times 1024$ pixels, and they occupy 62% of the execution time for the images composed of $2048 \times 2048$ pixels.

The performance loss caused by large datasets can be partially mitigated by adopting larger memory areas to store the image FFTs. As a final test, we processed 16384 images of $512\times 512$ pixels with the CPU–WITH_FT algorithm releasing to the program 23 GB of RAM. In this configuration, we obtained a speedup of a factor of 2 compared to the previous tests in which the RAM was limited to 8 GB, thanks to the larger available memory area. In fact, the image’s FFTs are recalculated six times by using 8 GB of RAM, but only two times by using 23 GB of RAM. We describe this test in more detail in “Appendix C”.

## Conclusion

In this article, we presented a new algorithm to calculate the structure function for image sets obtained by means of suitable optical techniques, like dynamic shadowgraphy, dynamic Schlieren, or differential dynamic microscopy. The algorithm is based on the temporal FFT of the image 2D spatial FFTs, rather than on differences of the latter. The software developed to implement the new algorithm has been tested against several other software available and it outperforms all of them by different factors depending on the image size and number.

In particular, we tested the new software with the one we developed a few years ago  [25]. While the old approach executes $\sim 30$ times faster in the GPU mode as compared to the CPU mode, the new method executes all the calculations within a similar amount of time on the GPU and the CPU. This result can be a valuable one for all the scientists that are not equipped with GPU hardware.

The increased performance in terms of time-saving is in itself a non-negligible advantage. However, the main reason for developing more performing software is to try to achieve real-time analysis of the images, so that the scientist can judge the quality of the measurement and thus modify the experimental parameters during the measurement itself. Analyzing with a delay of some hour means that the experiment must be performed again if the resulting data are not good in terms of signal-to-noise ratio or affected by other experimental issues.

The source code of the program developed in this work, which executes the algorithm both on CPU and GPU, is released under the GNU General Public License v.3 [34] and is freely available for download at [35]. The program can readily be used for calculating the structure function from an arbitrary set of images. A more efficient version of the code (about 10 times faster on GPU) is currently under development and will be commercially available in the next future.

## Figures and Tables

**Fig. 5:**
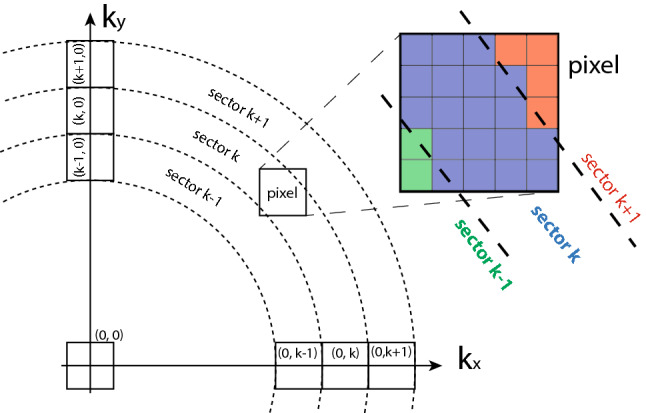
Pictorial representation of the azimuthal average scheme used to average the $d\left( m\right)$ matrices in the Fourier plane. A few pixels of the $d\left( m\right)$ are drawn as solid black squares on the plane. All the pixels that intersect a generic circular sector *k* are considered to compute the azimuthal average of a $d\left( m\right)$ matrix at the wavevector *k*. A generic pixel is enlarged to describe the antialiasing method that was adopted in the analysis. The subpixel having different colors are assigned to different wave vectors *k* in the azimuthal average as indicated by the color-coded labels. In the figure, we depict the configuration in which the pixel is subdivided in a $5 \times 5$ matrix, but we used an $8\times 8$ subdivision in the final analysis

**Fig. 6:**
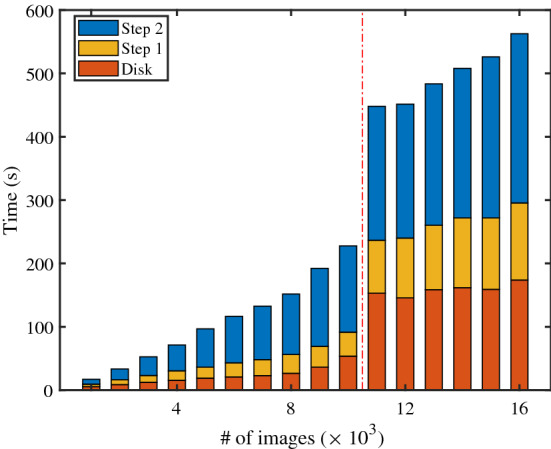
Execution time of the WITH_FT algorithm on CPU hardware on images of $512 \times 512$ pixels and limited RAM of 23 GB. The vertical line marks the crossing point in which the algorithm divides the execution from one into two groups. The labels “Step 1” and “Step 2” refer to the steps of the algorithm described in the main text and the label “Disk” refers to the memory I/O operations

**Fig. 7:**
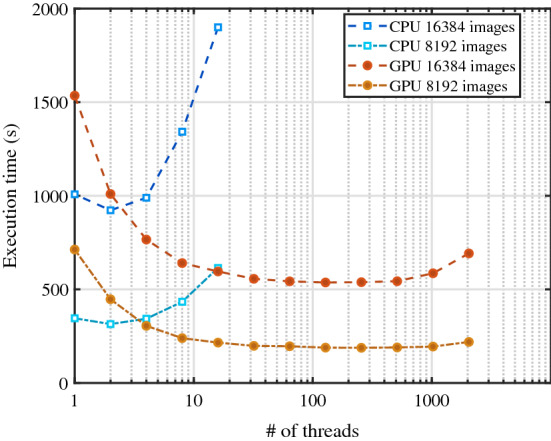
Execution time of the WITH_FT algorithm as a function of the different number of threads. The total number of images is indicated in the legend together with the hardware platform of execution. Image size is $512 \times 512$ pixels

**Fig. 8:**
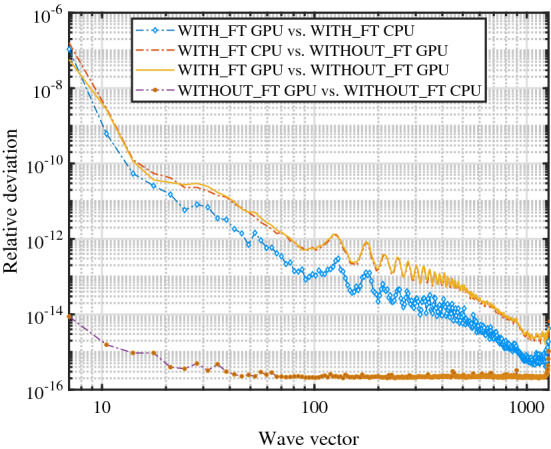
Relative deviation of the structure function values computed by using the different algorithms specified in the figure

## Data Availability

This manuscript has associated data in a data repository. [Authors’ comment: The source code of the program developed in this work is released under the GNU General Public License v.3 [34] and is freely available for download at [35]. The datasets analyzed during the current study are available from the corresponding author on reasonable request.]

## Appendix A: Azimuthal average

In this appendix, we describe how the azimuthal average of the structure function is calculated in our software program. Each $d\left( m\right)$ matrix is reduced by the average in a vector $\langle d\left( m\right) \rangle _\phi = f_k\left( m\right)$, where *k* is an integer number that indicates the amplitude of the wave vector. The average is performed over the pixels located at different circular sectors in the 2D-spatial-FFT plane as depicted in Fig. 5. We perform the average with an antialiasing algorithm that splits each pixel in an $8 \times 8$ matrix. Each sub-pixel is assigned its fractional position $\left( k_x, k_y\right)$ inside matrix so that its value can be averaged at the wave vector:

A.1 $$\begin{aligned} k = \left[ \,\sqrt{k_x^2+k_y^2}\,\right] \; , \end{aligned}$$

where “$\left[ \ldots \right]$” indicates the rounding operation.

## Appendix B: Analysis of the time sequences

In this appendix, we describe how we implemented the computation of the structure function on a single time sequence by using Eq. 2 in order to obtain the final result.

We split the calculation on the time sequence in two parts $d\left( m\right) = d_a\left( m\right) + d_c\left( m\right)$ where:

B.1 $$\begin{aligned} d_a\left( m\right)&=\frac{1}{N-m}\sum _{n=m}^{N-1}\left( |\tilde{I}_{n-m}|^2+|\tilde{I}_{n}|^2\right) \; , \end{aligned}$$

B.2 $$\begin{aligned} d_c\left( m\right)&=\frac{2}{N-m}\sum _{n=m}^{N-1}\text {Re}\left( \tilde{I^*}_{n-m}\tilde{I}_{n}\right) \; . \end{aligned}$$

The term $d_a\left( m\right)$, i.e., the average of the 2D spatial power spectra of the images with a computational complexity of $O\left( N\right)$, is calculated by using the following iterative formula:

B.3 $$\begin{aligned} d_a\left( N-n-1\right) =\frac{n}{n+1}d_a\left( N-n\right) +\frac{|I_{n}|^2+|I_{N-n-1}|^2}{n+1} \;, \end{aligned}$$

where the index *n* is in the range $\left[ 0,N-1\right]$.

The term $d_c\left( m\right)$ expresses the autocorrelation function of the time sequence of image FFTs. This second term is calculated by using the FFT in time taking advantage of the Wiener–Khinchin theorem. In this process we consider two requirements. First, the maximum performance gain obtainable by using the FFT algorithm is expected if the support points of the time sequences are a power of two. Second, the summation over *n* of Eq. B.2 takes into account only $N-m$ pairs of $\tilde{I}_{n}$ functions. These two requirements are incompatible with evaluating the FFT directly on *N* support points. The incompatibility emerges because the number *N* is not, in some cases, a power of two, and because the FFT algorithm imposes periodical boundary conditions on the time sequence. To meet both requirements, we zero-padded the time sequences to $N_2$ support points, where $N_2$ is given by:

B.4 $$\begin{aligned} \log _2{N_2} = \lceil \log _2{\left( N\right) }\rceil + 1 \; , \end{aligned}$$

where “$\lceil \ldots \rceil$” denotes the ceiling operation. This padding operation allows us to take advantage of the FFT speed-up, and calculate exactly Eq. 2, without any influence caused by the periodical boundary conditions. The calculation of $d_c\left( m\right)$ for a single time sequence can be broken down into the following operations.

- The time sequence is zero-padded to $N_2$ complex support points.
- The padded sequence is Fourier-transformed by FFT in time.
- Each element of the FFT is squared in modulus obtaining the power spectrum of the time sequence.
- The inverse FFT is applied to the power spectrum.
- The new vector is truncated to *N* support points and the imaginary part is discarded.
- The resulting sequence is the autocorrelation of the time sequence and it is normalized by the ramp vector $1/\left( N-m\right)$.

Finally, $d_a\left( m\right)$ and $d_c\left( m\right)$ are added together to obtain the structure function $d\left( m\right)$.

## Appendix C: Comparison with other software

**Table 1:** Execution times of the programs Soft_1, Soft_2, Soft_3, GPU-WITHOUT_FT (label “NO_FT”), GPU-WITH_FT (label “GPU”) and CPU-WITH_FT (label “CPU”)

N	Time (s)					
	Soft_1	Soft_2	Soft_3	NO_FT	GPU	CPU
64	24	9	6	$<1$	12	1
128	70	17	11	1	14	2
256	246	35	51	3	16	4
512	923	80	113	9	20	8
1024	3516	181	215	31	27	17
2048	14494	430	630	113	38	35
4096	51343	1022	1370	446	86	117
8192	$>10^5$	2091	2889	1740	190	314
16384	$>10^5$	4638	5783	6921	574	922

The label “N” indicates the number of images. For the comparison, we used images composed by $512 \times 512$ pixels

In this appendix, we compare the execution time of our algorithm with other software programs that calculate the structure function. For the comparison, we used the programs specified in refs. [25, 30–32]. The program of ref. [25] is already introduced in the main text with the name of GPU-WITHOUT_FT. We will refer to the software reported in [30] as Soft_1, the software reported in [31] as Soft_2 and the software reported in [32] as Soft_3. Soft_1 and Soft_2 are written in Matlab and Soft_3 is written in Python. We benchmarked the execution time of all the programs against each other by analyzing images of $512 \times 512$ pixels.

In Table 1, we present the total execution time of the different software to complete the calculation of the structure function as a function of the image number. We see that the GPU-WITHOUT_FT algorithm executes faster or within comparable times compared to Soft_1-3 and, thus, was selected as the software for comparison in the main text.

We see that, compared to Soft_1-3, the new program speeds up the calculation by a factor larger than 10 while processing more than 1024 images. For example, the WITH_FT algorithm is about 415, 12, 18 times faster than Soft_1, Soft_2 and Soft_3, respectively, at processing 2048 images for both versions CPU and GPU and about 3 times faster than the GPU-WITHOUT_FT.

## Appendix D: Group execution

The program described in this work splits the calculations into groups if the data of all wave vector components for all the images exceeds the available storage memory. The method WITHOUT_FT uses a first-in-first-out (FIFO) memory scheme already described in ref. [25]. This approach aims to calculate groups of complete $d\left( m\right)$ matrices. The WITH_FT algorithm, instead, operates sequentially on different groups of wave vectors for all the $d\left( m\right)$ and saves the partial results of each group on the hard drive. The partial results are merged at the final stage of the program. In practice, in both algorithms, the images are loaded and Fourier transformed one time for each group because only a part of the FFTs data can be saved on the local memory. The impact of repeating these operations over the entire execution time is presented in Fig. 6, in which we present the execution time as a function of the number of images to process. In this test, we executed the WITH_FT algorithm on CPU hardware with images of $512 \times 512$ pixels by releasing to the program 23 GB of RAM. In the figure, the vertical red line marks the crossing point from one-group to two-group execution. We see that the time spent by the program in memory operations and step one suddenly doubles by crossing the line. This happens because the bidimensional FFTs of the images and the corresponding I/O operations must be executed two times instead of only one.

## Appendix E: Number of threads

The WITHOUT_FT algorithm executes efficiently with a parallel computing scheme on GPU hardware which makes use of all the available CUDA-threads. This is not the case for the WITH_FT scheme. To analyze the influence of parallel computing on the execution time of the WITH_FT algorithm, we implemented the WITH_FT method with a user-configurable number of threads both in the CPU mode and the GPU mode. The number of threads in the CPU mode refers to the number of threads spawned to execute a particular task, such as the FFT operations. In the GPU mode, the number of threads selects the amount of CUDA threads of each CUDA kernel. In both CPU and GPU modes, the number of threads also determines the number of time sequences that are processed in parallel. Figure 7 presents the total execution times of the program as a function of the different number of threads for 8192 and 16384 images. In the test, we selected images composed of $512 \times 512$ pixels. Parallel computing achieves a minimal or detrimental impact on the speed-up factor in the CPU mode. In the GPU mode, the performance gain saturates at around 32 threads, with a peak performance at 256 GPU threads. Based on this result, we selected the optimal number of two CPU threads and 256 GPU threads for all other tests presented in this work.

## Appendix F: Numerical deviations

In this appendix, we compare the numerical discrepancies of the program emerging from the execution of the DDM analysis by using different algorithms and hardware platforms. The deviations in the calculated structure functions are caused by the different numerical approaches and adopted libraries, each of which introduces different numerical errors. To quantify these discrepancies, we compared the analysis results of the data presented in Fig. 1 obtained by the four execution modes of the program. To quantify the deviations, we calculated the relative deviation $\delta$ as a function of the wave vector *k* for the azimuthal averages of the structure function. We define the deviation of the structure functions as:

F.1 $$\begin{aligned} \delta _{k}=\frac{2}{N}\sum _m \left|\frac{f_k\left( m\right) -g_k\left( m\right) }{f_k\left( m\right) +g_k\left( m\right) }\right|, \end{aligned}$$

where *m* refers to the time delay in the range [1, 2000] and $f_k(m)$ and $g_k(m)$ are the azimuthal averages of the *d*(*m*) matrices using different algorithms. Figure 8 shows the value of $\delta$ as a function of the wave vector *k*. The relative deviation between GPU and CPU in the case of WITHOUT_FT is always less than $10^{-14}$, and decreases for increasing wave vectors. A similar decreasing trend as a function of the wave vectors is also visible for the WITH_FT algorithm, even though the relative deviations at small wave vectors are approximately $10^{-7}$. We also note that the relative uncertainty shows an oscillatory trend, similar to the one visible in the structure function, but with an opposite phase.
